# Effects of deep tillage combined with organic amendments application on carbon and nitrogen storage within aggregates and wheat yield

**DOI:** 10.3389/fpls.2025.1669580

**Published:** 2025-11-28

**Authors:** Wenlong Cheng, Rongyan Bu, Shang Han, Shan Tang, Hui Wang, Min Li, Rui Zhu, Fahui Jiang, Mengmeng Tang, Ji Wu

**Affiliations:** Soil and Fertilizer Institute, Anhui Academy of Agricultural Sciences (National Agricultural Experimental Station for Soil Quality, Taihe)/Anhui Provincial Key Laboratory of Nutrient Cycling and Arable Land Conservation, Hefei, Anhui, China

**Keywords:** carbon, nitrogen, wheat, straw retention, manure addition

## Abstract

**Introduction:**

Soil organic carbon (SOC) and total nitrogen (TN) stocks are key determinants of the productive capacity of agricultural soils. This study aims to elucidate the effects of long-term tillage practices combined with organic amendments on SOC and TN sequestration within soil aggregates and their subsequent relationship with wheat yield.

**Methods:**

A six-year field experiment (initiated in 2016) was conducted, evaluating the following treatments: rotary tillage with straw, rotary tillage with manure, rotary tillage with straw plus manure, deep tillage with straw, deep tillage with manure and deep tillage with straw plus manure.

**Results:**

Relative single organic amendments, both rotary and deep tillage with straw plus manure significantly enhanced macro-aggregate-associated SOC and TN stocks in the 0–15 cm layer by 7.86–23.29% and 16.36–18.99%, respectively. In the 15–30 cm layer, deep tillage with single organic amendments increased macro-aggregate-associated SOC and TN contents by 7.56–18.81% and 5.29–21.47%, respectively compared with rotary tillage. However, deep tillage also reduced macro-aggregate proportions by 5.36–8.16%, which decreased associated SOC and TN stocks by 6.12–7.87% and 6.99–8.53%, respectively. In contrast, deep tillage with straw plus manure increased macro-aggregate-associated SOC and TN contents (18.42–19.39% and 10.17–12.76%, respectively) without reducing macro-aggregate proportions, thereby enhancing SOC and TN stocks in macro-aggregates by 10.49–26.89% and 9.07–26.32%, respectively.

**Discussion:**

These findings indicate that the enrichment of subsoil macro-aggregate-associated SOC and TN strongly correlated with higher wheat yield, demonstrating that deep tillage with straw and manure is an effective practice for improving soil health and sustaining productivity.

## 1 Introduction

Healthy soil underpins farmland productivity, food security, ecological sustainability, and ultimately human well-being (Bouma, 2014; Keesstra et al., 2016). Soil organic carbon (SOC) and total nitrogen (TN) are fundamental indicators soil health, as their quantity and distribution strongly regulate soil quality, cropland productivity and yield stability (Mo et al., 2019). Soil aggregates contribute the structural basis of soils and act as the primary carriers of carbon and nitrogen reserves. Soil aggregates are typically divided into macroaggregates and microaggregates using a size boundary of 0.25 mm (Wang et al., 2023). The macroaggregates provide physical protection for organic matter by encasing it within their structure, thereby slowing its decomposition and promoting the stabilization of soil organic carbon (SOC) (Wang et al., 2023). Furthermore, they create a favorable pore structure that enhances water infiltration, aeration, and root penetration (Tian et al., 2022). In contrast, microaggregates, while highly stable, form the building blocks of macroaggregates and are associated with longer-term carbon storage but offer less immediate nutrient cycling potential (Lehmann and Kleber, 2015). The distribution of soil carbon and nitrogen in macro-aggregates particle sizes directly influences soil carbon sequestration and nitrogen cycling process (Guo et al., 2010; Kan et al., 2020; Li et al., 2021). Tillage practices, particularly when combined with organic matter return, play a critical role in shaping the distribution of SOC and TN across aggregate fractions within i (Andruschkewitsch et al., 2013; Song et al., 2016).

Deep tillage is often considered superior to conventional rotary tillage due to its capacity to break compacted plow pans, increase effective topsoil depth, and modify soil physical conditions (Feng et al., 2020; Scanlan and Davies, 2019). However, deep tillage disrupts a greater soil volume, which can hinder macro-aggregates (Ashagrie et al., 2007; Li et al., 2024). Furthermore, mixing nutrient-poor subsoil with topsoil. under deep tillage may dilute soil fertility, accelerate organic matter mineralization, and potential reduce crop yields. To counter these adverse effects, additional exogenous organic inputs are required to sustain soil fertility under deep tillage conditions. Long-term input of organic amendments, such as crop straw and animal manure, has been shown to stimulate microbial activity, enhance aggregate composition, and increased associated SOC and TN stocks (Tian et al., 2022). Moreover, organic inputs strengthen aggregate stability by promoting the cementation of soil particles through organic and inorganic binding agents (Kiattisak and Akira, 2023), while simultaneously contributing to yield improvement (Kong et al., 2024). Thus, the judicious integration of organic amendments with tillage practices may offer an effective pathway to mitigate the drawbacks of deep tillage. However, it remains unclear how the combined treatments of different tillage depths and organic amendments affect aggregate composition, carbon and nitrogen storage, and wheat yield across different soil layers.

Based on this understanding, we hypothesized that: 1) deep tillage would decrease the proportion of soil macro-aggregates compared to rotary tillage, thereby lowering SOC and TN stocks in this fraction and reducing wheat yield; and 2) the extent of SOC and TN enhancement in macro-aggregates, as well as yield provement, would depend on the type of organic amendment and its interaction with tillage. To test these hypotheses, a long-term field experiment was conducted combining two tillage practices (deep tillage, rotary tillage) with three organic amendments strategies (straw return, manure application, and straw plus manure). The objective was to elucidate the interactive effects of tillage and organic amendments on soil aggregate-associated SOC and TN stocks, as well as wheat yield. Findings from this study are expected to provide empirical evidence supporting soil health management strategies that simultaneously enhance soil quality and sustain high wheat productivity.

## 2 Materials and methods

### 2.1 Experimental site

**Figure 1:**
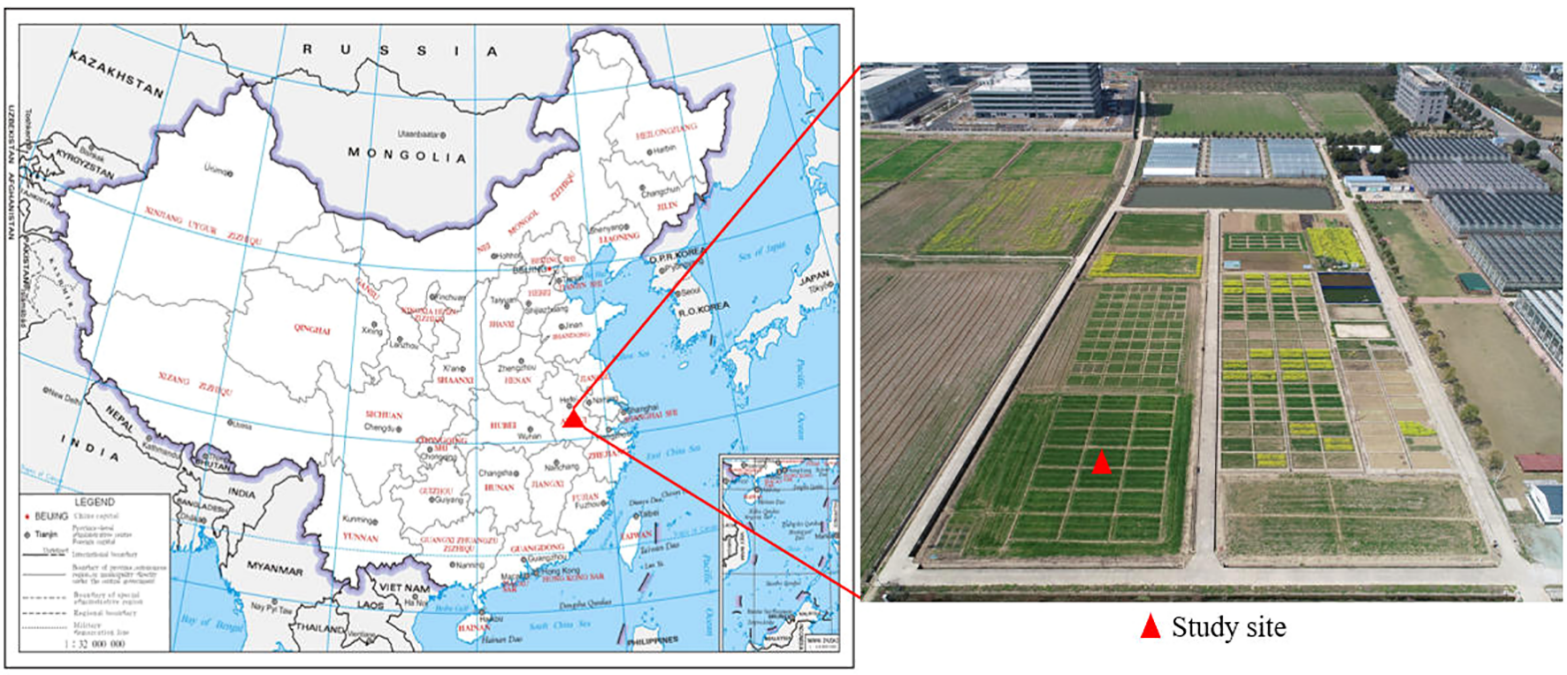
Location of the study sites in Lu’an, Anhui Province, China.

The field experiment (**Figure 1**) was conducted in Lu’an, Anhui Province, China (31°01′36″ N, 116°26′65″ E), a region characterized by a typical northern subtropical monsoon climate. The site has a mean annual precipitation of 1149.1 mm, an average sunshine duration of 1803.5 h, and a mean annual temperature of 15.8 °C. The prevailing cropping system is a rice-wheat rotation. The soil at the site is classified as paddy soil (Hydragric Anthrosols), derived from river alluvium deposits, with a clay loam texture and categorized as Stagnic Anthrosol according to FAO (2015). The initial chemical properties of the surface soil (0–20 cm) is provided in **Table 1**.

**Table 1:** Chemical properties of the experimental soil.

Soil layer (cm)	pH	Organic carbon (g/kg)	Total N (g/kg)	Olsen-P (mg/kg)	Available K (mg/kg)
0–15	5.96	13.83	1.44	12.76	107.42
15–30	6.57	7.80	0.90	9.56	94.79

### 2.2 Experimental design

The experiment was established in October 2016 and consisted of six treatments: rotary tillage with straw retention (RS), rotary tillage with manure addition (RM), rotary tillage with straw plus manure application (RSM), deep tillage with straw retention (DS), deep tillage with manure addition (DM), and deep tillage with straw plus manure application (DSM). The treatments were arranged in a randomized complete block design with three replicates. Each plot measured 60 m^2^ each (6 m × 10 m). Rotary tillage was performed at a depth of 15 cm, and deep tillage at 30 cm, using a model 170 tillage machine (Zhongqi Machinery Sales Company, Qufu, China). Tillage was conducted prior to the planting of crop. For straw retention treatments, rice and wheat residues were chopped to 10 cm length and incorporatedafter harvest. The total carbon and nitrogen inputs for each treatments in **Table 2**.

**Table 2:** The total carbon and nitrogen inputs for each treatment during 2016–2022 (kg ha^−1^).

C and N inputs	Treatment	Fertilizers		Straw				Manure	Sum
		Rice	Wheat	Rice		Wheat			
				Range	Mean	Range	Mean		
C	RS	/	/	2885–3110	3037	1405–1774	1656	/	4693
	DS	/	/	2795–3031	2889	1392–1565	1515	/	4404
	RM	/	/	/	/	/	/	1552.5	1552.5
	DM	/	/	/	/	/	/	1552.5	1552.5
	RSM	/	/	3090–3406	3187	1679–1914	1814	1552.5	6553.5
	DSM	/	/	3146–3536	3427	1877–2047	1956	1552.5	6935.5
N	RS	210	180	56.9–61.3	59.9	15.8–20.0	18.6	/	468.5
	DS	210	180	55.1–59.8	57.0	16.4–18.5	17.9	/	464.9
	RM	210	180	/	/	/	/	100.4	490.4
	DM	210	180	/	/	/	/	100.4	490.4
	RSM	210	180	60.1–66.3	62.0	19.2–21.9	20.8	100.4	573.2
	DSM	210	180	61.2–68.8	66.7	21.5–23.5	22.4	100.4	579.5

RS, RM, and RSM represent rotary tillage with straw, manure, and straw plus manure application, respectively. DS, DM, and DSM represent deep tillage with straw, manure, and straw plus manure application, respectively. Error bars represent standard deviation of the mean (n = 3). Different lowercase letters indicate significant differences between the treatments at p< 0.05 by the ANOVA, LSD.

Chemical fertilizers were applied uniformly across all treatments. During the rice season, application rates were 210 kg N ha^-1^, 75 kg P_2_O_5_ ha^-^¹, and 120 kg K_2_O ha^-^¹. Nitrogen (as urea, 46% N) was applied in three splits: 50% as basal fertilizer, 30% at tillering, and 20% at heading. Phosphorus (as granular calcium phosphate, 12% P_2_O_5_) was applied once as basal fertilizer, and potassium (as potassium chloride, 60% K_2_O) was applied in two splits: 70% as basal and 30% at heading. In the wheat season, chemical fertilizer inputs were 180, 60, and 90 kg ha^-1^ for N, P_2_O_5_, and K_2_O. Nitrogen was applied in two splits: 60% as basal and 40% at jointing. Phosphorus and potassium were applied entirely as basal fertilizers. Wheat (Triticum aestivum L., cv. ‘Yangmai 25’) was sown in early November at a seeding rate of 225 kg ha^-^¹ and harvested in late May.

### 2.3 Sample collection and testing

wheat was harvested from each plot at maturity each year to determine grain yield. Grains were threshed and weighed, and grain moisture content was measured to adjust yield to a standard moisture basis. In 2022, following harvest, soil samples were collected from each plot at depths of 0–15 cm and 15–30 cm using the five-point sampling method (Liu et al., 2019). At each depth, five cores were taken and composited into one homogenized sample per plot. Soil bulk density was measured at both depths using the steel cylinder method with a 100 cm³ core (Zhai et al., 2021). In the laboratory, samples were air-dried after removing visible plant residues and gravel. Soil clods were gently broken along natural planes before aggregate fractionation. Aggregate size distribution was determined by the wet-sieving method (Elliott, 1986). Briefly, 50 g of air-dried soil was pre-soaked in deionized water for 24 h and then subjected to wet sieving using a set of nested sieves (2, 0.25, and 0.053 mm) on a soil aggregate analyzer (Shunlong Laboratory Instruments Factory, Shangyu, China) oscillating at 20 cycles min^-^¹ with a 4 cm amplitude for 10 min. Four aggregate size classes were obtained: >2 mm, 2–0.25 mm, 0.25–0.053 mm, and<0.053 mm. Fractions were collected, oven-dried at 60°C for 10 h, and weighed. Aggregate stability indices, including mean weight diameter (MWD) and geometric mean diameter (GMD), were calculated following standard procedures (Liu et al., 2014). Each aggregate fraction was finely ground and passed through a 100-mesh sieve prior to chemical analyses. SOC was determined by the potassium dichromate oxidation method, and TN was measured by the Kjeldahl method (Bao, 2000).

### 2.4 Data analysis

Soil bulk density (Equation 1), mean weight diameter (MWD) (Equation 2), geometric mean diameter (GMD) (Equation 3), and soil aggregate-associated SOC (Equation 4) and TN (Equation 5) stocks were calculated using the following equations (Eynard et al., 2005; Liu et al., 2014; Dai et al., 2024; Zhai et al., 2021):

(1) $$B_{d}=\frac{\left(W_{2}-W_{1}\right)\times \left(1-W\%\right)}{V}$$

(2) $$\;MWD=\sum_{i=1}^{n}X_{i}W_{i}$$

(3) $$GMD=\text{exp}\left(\frac{\sum_{i=1}^{n}W_{i}ln\bar{X_{i}}}{\sum_{i=1}^{n}W_{i}}\right)$$

(4) $$S_{c}=W_{i}\times C_{i}\times B_{d}\times H\times \;10$$

(5) $$S_{n}=W_{i}\times N_{i}\times B_{d}\times H\times \;10$$

where Bd is the soil bulk density (g cm^-^³); W_1_ is the weight of the cutting ring (g); W_2_ is the combined weight of soil and cutting ring (g); W% is the gravimetric water content of fresh soil samples (%); V is the volume of the cutting ring (cm³); X_i_ is the mean diameter of aggregate fraction i (mm); W_i_ is the proportion of aggregate fraction i (%); Sc and Sn are the SOC and TN stocks in a given aggregate fraction (kg ha^-^¹), respectively; C_i_ and N_i_ are the SOC and TN concentrations in fraction i (g kg^-^¹); and H is the thickness of the soil layer (cm). All data were subjected to statistical analysis using SPSS 20.0 (IBM Corp., Armonk, NY, USA) and Excel 2010 (Microsoft Corp., Redmond, WA, USA). Treatment means were compared using the least significant difference (LSD) test at p< 0.05. Graphical outputs were generated with OriginPro 2018 (OriginLab Corp., Northampton, MA, USA). To explore the contribution of aggregate-associated SOC and TN stocks to wheat yield, principal component analysis (PCA) was performed using OriginPro 2018. In addition, a random forest model was applied to quantify the relative importance of SOC and TN stocks across different aggregate fractions in predicting wheat yield. The relationships between wheat yield and SOC/TN stocks were further examined using linear regression and Pearson correlation analysis.

## 3 Results

### 3.1 Effect of tillage and organic amendments on oil aggregates composition

**Figure 2:**
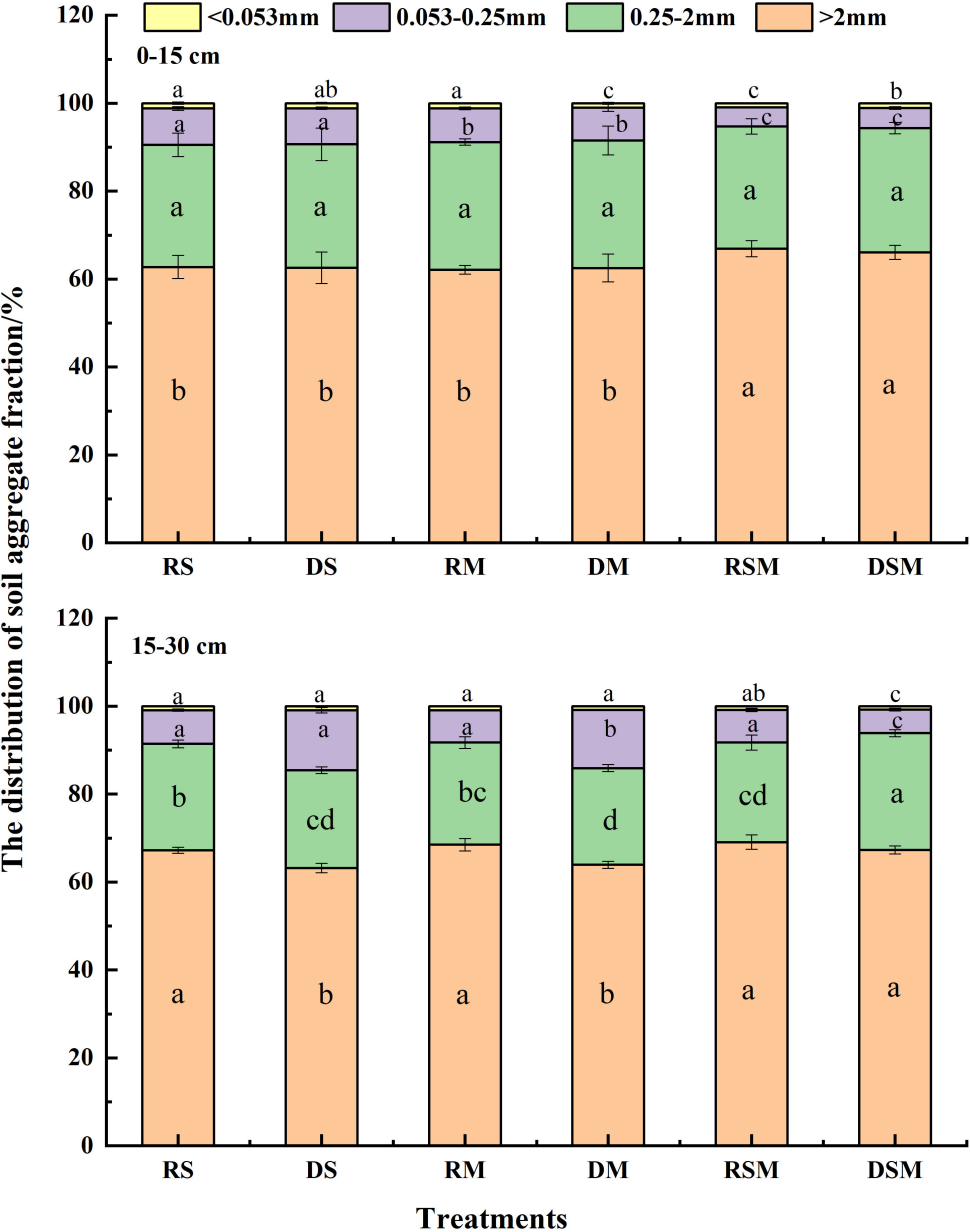
Soil aggregate composition under different tillage practices and organic inputs. RS, RM, and RSM represent rotary tillage with straw, manure, and straw plus manure application, respectively. DS, DM, and DSM represent deep tillage with straw, manure, and straw plus manure application, respectively. Error bars represent standard deviation of the mean (n = 3). Different lowercase letters indicate significant differences between the treatments at *p*<0.05 by the ANOVA, LSD.

In the 0–15 cm soil layer (**Figure 2**), the combined application of straw and manure (RSM, DSM) markedly improved soil aggregate distribution compared with single organic amendments (RS, RM, DS, DM). Specifically, the proportion of macroaggregates (>2 mm) increased by 5.56–7.79%, while the proportions of microaggregates (0.053–0.25 mm) and silt–clay fractions (<0.053 mm) decreased by 44.28–47.25% and 17.25–18.93%, respectively.

In the 15–30 cm soil layer, deep tillage with single organic amendments (DS, DM) significantly reduced the proportions of >2 mm and 0.25–2 mm aggregates by 6.00–6.69% and 5.36–8.16%, respectively, compared with rotary tillage (RS, RM). Conversely, the proportion of 0.053–0.25 mm aggregates increased substantially by 77.91–79.14% under deep tillage. However, when straw was combined with manure under deep tillage (DSM), these negative effects were mitigated: the proportion of >2 mm aggregates was maintained, and the proportion of 0.25–2 mm aggregates was 17.37% higher than under rotary tillage with straw plus manure (RSM). Furthermore, DSM significantly reduced the proportions of 0.053–0.25 mm and<0.053 mm fractions by 27.81% and 9.43%, respectively, relative to RSM.

**Figure 3:**
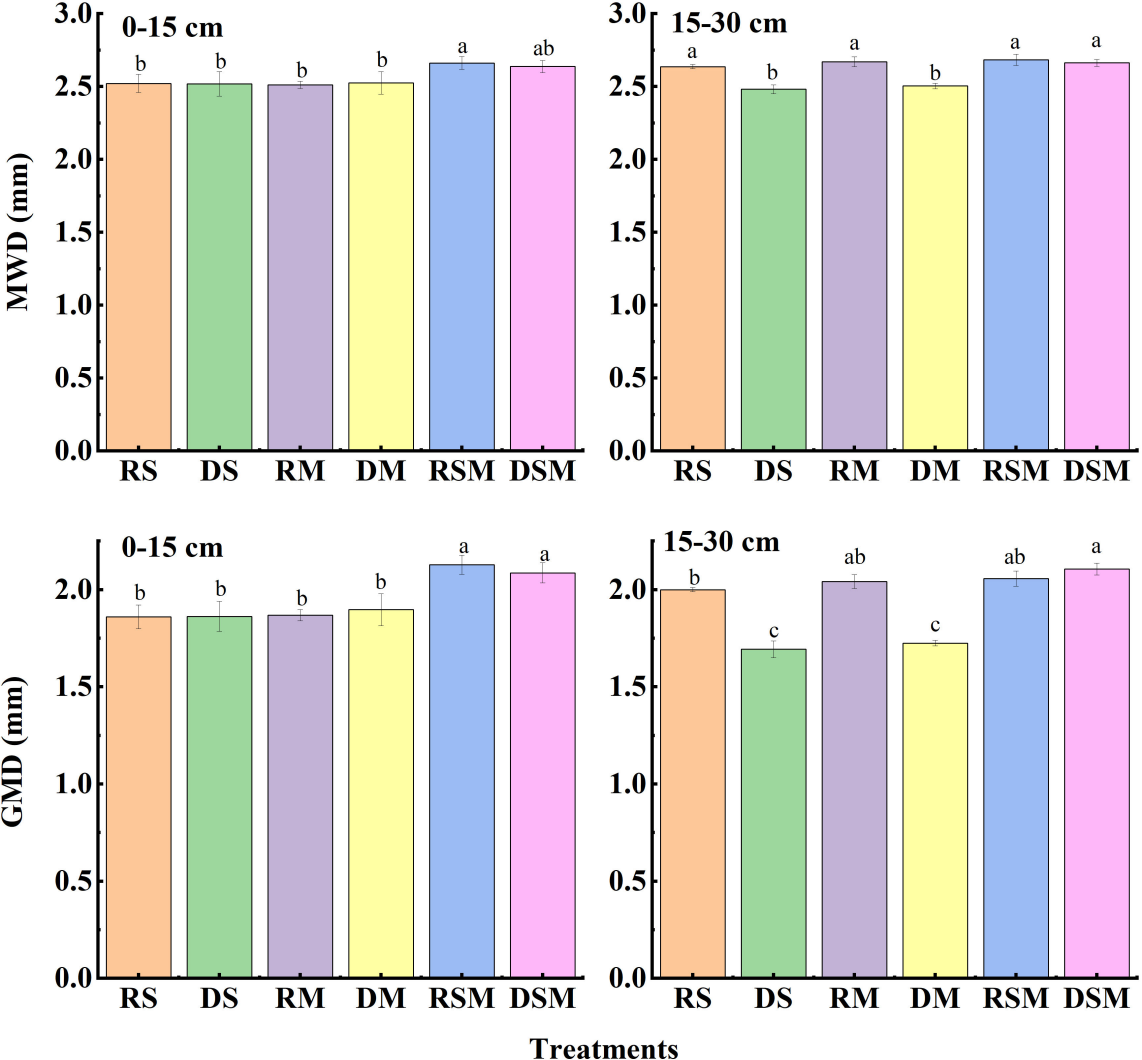
Mean weight diameter (MWD) and geometric mean diameter (GMD) of soil aggregates under different treatments. RS, RM, and RSM represent rotary tillage with straw, manure, and straw plus manure application, respectively. DS, DM, and DSM represent deep tillage with straw, manure, and straw plus manure application, respectively. Error bars represent standard deviation of the mean (n = 3). Different lowercase letters indicate significant differences between the treatments at *p*<0.05 by the ANOVA, LSD.

**Figure 4:**
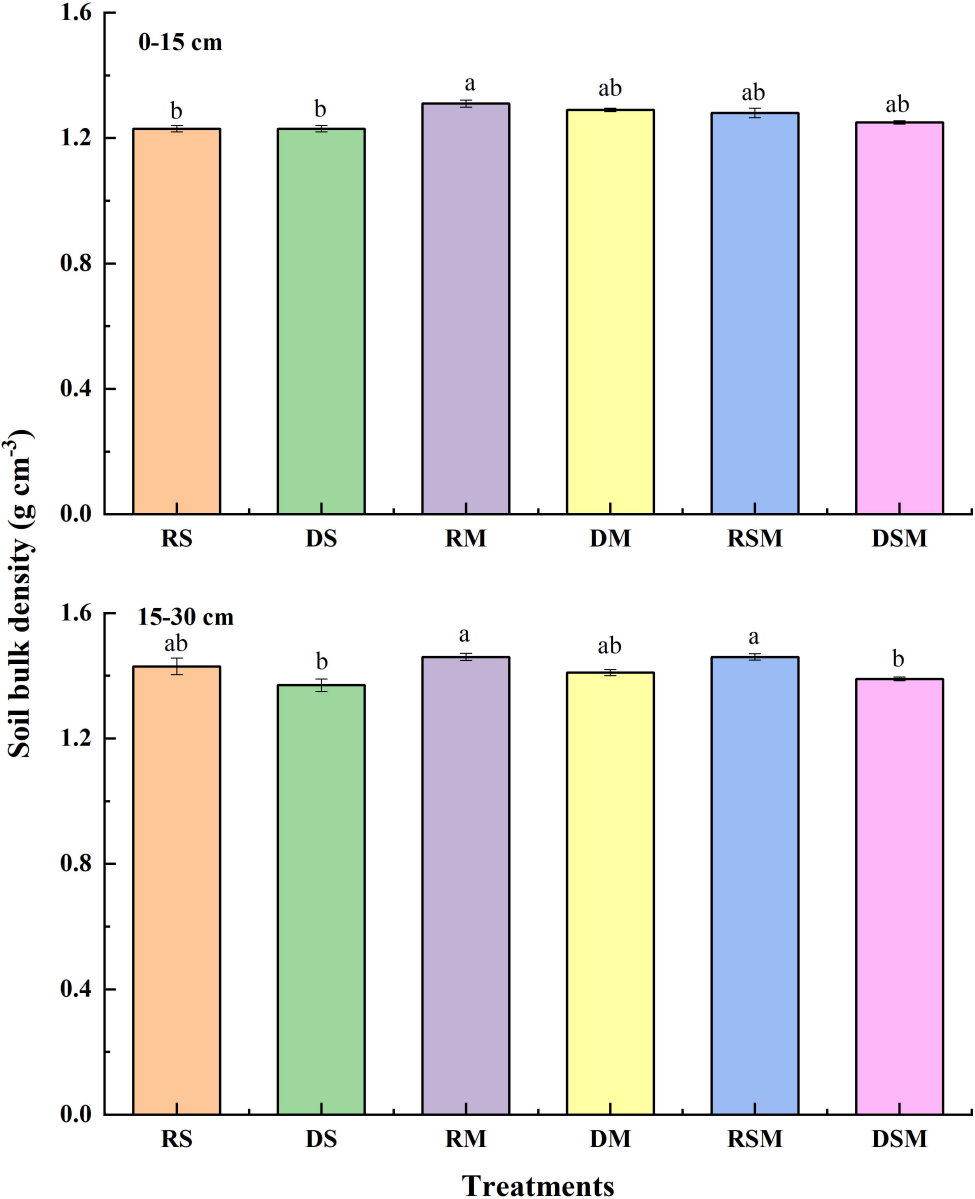
Soil bulk density under different treatments. RS, RM, and RSM represent rotary tillage with straw, manure, and straw plus manure application, respectively. DS, DM, and DSM represent deep tillage with straw, manure, and straw plus manure application, respectively. Error bars represent standard deviation of the mean (n = 3). Different lowercase letters indicate significant differences between the treatments at *p*<0.05 by the ANOVA, LSD.

The stability indices of soil aggregates (**Figure 3**) showed similar trends. In the 0–15 cm layer, geometric mean diameter (GMD) increased by 9.98–14.44% under RSM and DSM relative to other treatments. In the 15–30 cm layer, deep tillage with single organic amendments (DS, DM) reduced mean weight diameter (MWD) and GMD by 15.50–15.69% and 6.06–6.37%, respectively, compared with rotary tillage (RS, RM). Notably, these reductions were not observed under straw plus manure application, indicating that combined organic amendments buffered the adverse impact of deep tillage on aggregate stability (**Figure 4**). In the 0–15 cm layer, straw return treatments (RS, DS) reduced bulk density by 6.50% compared with manure-only application (RM). In the 15–30 cm layer, deep tillage combined with straw plus manure (DSM) significantly decreased bulk density by 5.04% compared with rotary tillage (RSM).

### 3.2 Effect of tillage and organic amendments on soil aggregate-associated carbon and nitrogen

**Figure 5:**
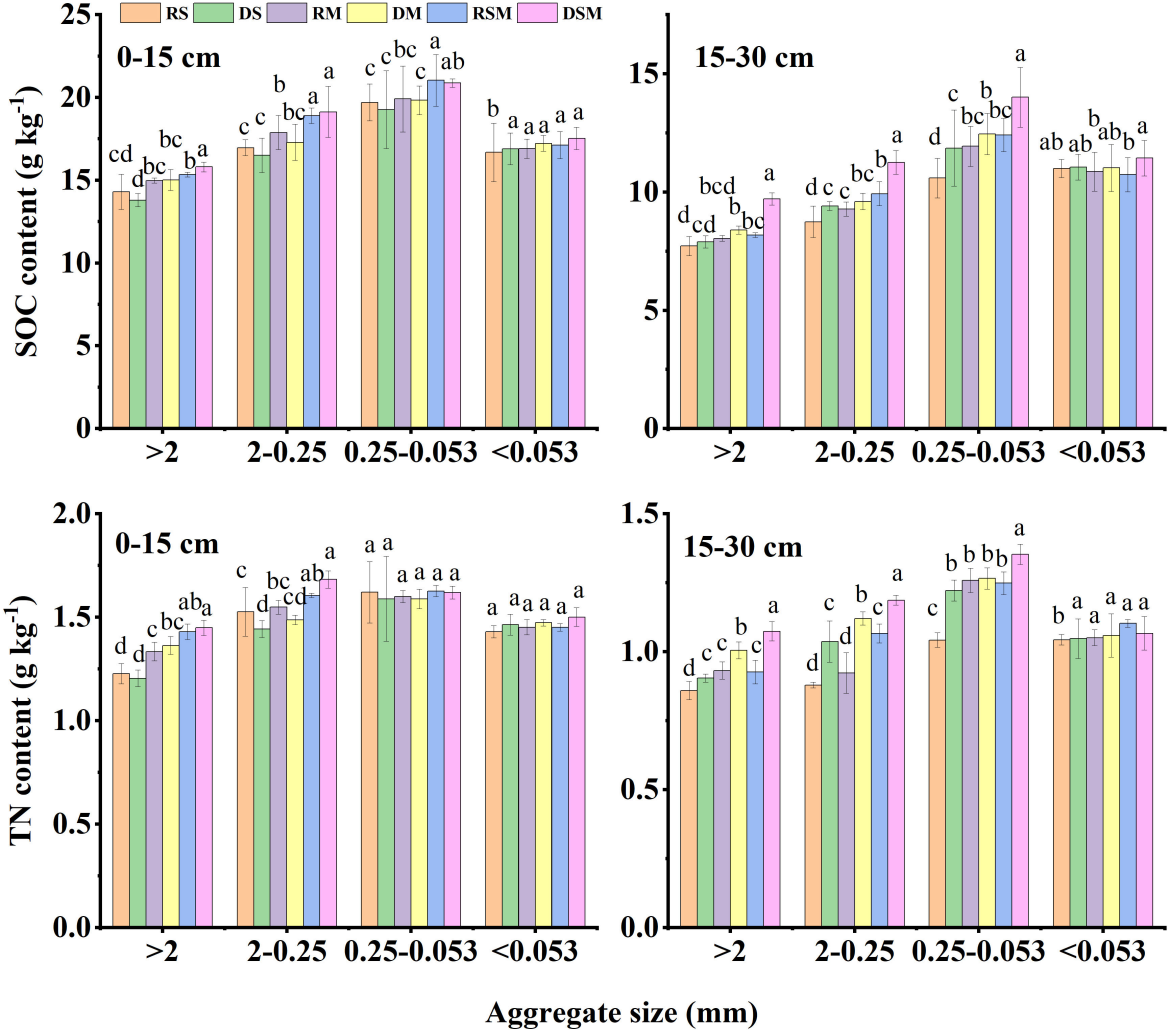
Soil organic carbon (SOC) and total nitrogen (TN) contents in various soil aggregate fractions under different treatments. RS, RM, and RSM represent rotary tillage with straw, manure, and straw plus manure application, respectively. DS, DM, and DSM represent deep tillage with straw, manure, and straw plus manure application, respectively. Error bars represent standard deviation of the mean (n = 3). Different lowercase letters indicate significant differences between the treatments at *p*<0.05 by the ANOVA, LSD.

Soil organic carbon (SOC) and total nitrogen (TN) contents associated with aggregates, particularly in fractions >0.053 mm, were markedly influenced by tillage and organic amendments (**Figure 5**). Compared with single organic amendments, the combined application of straw and manure significantly enhanced SOC and TN contents in macroaggregates (>2 mm and 0.25–2 mm), with increases of 5.26–23.06% and 5.14–21.25%, respectively, across both soil layers. Similarly, in the 0–30 cm soil profile, SOC and TN contents in 0.053–0.25 mm aggregates increased by 5.63–18.13% and 6.89–19.76%, respectively, under straw plus manure relative to single amendments.

Deep tillage also promoted SOC and TN accumulation compared with rotary tillage, particularly in the 15–30 cm soil layer. SOC and TN contents in macroaggregates increased by 7.56–18.81% and 5.29–21.47%, respectively, under deep tillage. In the same layer, SOC and TN contents of 0.053–0.25 mm aggregates were elevated by 11.97–12.86% and 8.36–17.24%, respectively. Among all treatments, deep tillage with straw plus manure (DSM) exerted the strongest positive effect on SOC and TN sequestration. In the 0–15 cm layer, DSM increased SOC contents in >2 mm and 0.25–2 mm aggregates by 9.90% and 13.25%, and TN contents by 13.29% and 14.88%, respectively, compared with other treatments. In the 15–30 cm layer, DSM further enhanced SOC contents in >2 mm and 0.25–2 mm aggregates by 19.39% and 18.42%, respectively, along with corresponding TN increases of 12.76% and 10.17%.

### 3.3 Effects of tillage and organic amendments on soil aggregate-associated carbon and nitrogen stocks

Soil aggregate-associated SOC and TN stocks responded significantly to both tillage depth and organic amendment type (**Tables 3**, **4**). In the 0–15 cm soil layer, the combined application of straw and manure markedly increased SOC stocks in >2 mm and 0.25–2 mm aggregates by 7.86–23.29% and 16.36–18.99%, respectively, relative to single organic amendments. Conversely, SOC stocks in 0.053–0.25 mm aggregates and<0.053 mm clay fractions declined by 37.27–42.28% and 13.65–17.96%, respectively. TN stocks followed a similar trend, with increases of 8.86–29.51% and 7.05–19.56% in >2 mm and 0.25–2 mm aggregates, but substantial reductions of 39.46–45.30% and 13.85–19.19% in 0.053–0.25 mm aggregates and<0.053 mm clay fractions, respectively.

**Table 3:** Soil aggregate-associated organic carbon stocks under different treatments (kg ha^−1^).

Soil layer (cm)	Treatment	Aggregate size (mm)				
		>2	0.25–2	0.053–0.25	<0.053	Sum
0–15	RS	16537.8±917. 6c	8691.1±692.9c	3000.4±184.8ab	364.5±101.6b	28593.8±1074.0bc
	DS	15920.6±585.0c	8524.4±969.8c	2924.3±403.8ab	350.3±59.2bc	27719.6±918.1c
	RM	18332.3±369.1 b	10253.1±841.9a	3020.7±436.6a	384.6±17.8a	31990.7±1335.2a
	DM	18191.7±440.2 b	9695.7±844.0b	2865.5±211.8b	337.6±66.8c	31090.5±170.8a
	RSM	19772.3±533.9a	10113.0±591.7ab	1743.6±117.1c	315.5±31.2d	31944.4±539.9a
	DSM	19628.0±193.1a	10142.8±408.1ab	1797.7±119.4c	356.5±11.1b	31925.0±492.3a
15–30	RS	11129.9±651.7c	4542.3±324.4c	1749.4±230.8c	211.7±22.3a	17633.3±903.5d
	DS	10253.8±482.6d	4299.5±59.6d	3314.0±285.5a	210.4±26.9a	18077.6±768.2cd
	RM	12087.0±228.4b	4738.3±346.4bc	1932.8±170.8b	213.5±11.6a	18971.7±116.6bc
	DM	11347.3±122.9 c	4464.9±123.9d	3479.3±233.5a	206.2±11.9a	19497.8±247.4b
	RSM	12371.1±154.4b	4925.9±477.3b	2018.8±162.1b	200.9±15.3a	19516.7±303.6b
	DSM	13668.9±524.5a	6250.3±288.3a	1571.4±188.4d	184.9±9.3b	21675.5±99.7a

RS, RM, and RSM represent rotary tillage with straw, manure, and straw plus manure application, respectively. DS, DM, and DSM represent deep tillage with straw, manure, and straw plus manure application, respectively. Error bars represent standard deviation of the mean (n = 3). Different lowercase letters indicate significant differences between the treatments at p< 0.05 by the ANOVA, LSD.

**Table 4:** Soil aggregate-associated total nitrogen stocks under different treatments (kg ha^−1^).

Soil layer (cm)	Treatment	Aggregate size (mm)				
		>2	0.25–2	0.053–0.25	<0.053	Sum
0–15	RS	1423.8±121.0c	782.9±91.7c	246.6±18.3a	31.0±6.5b	2484.3±100.8b
	DS	1389.9±50.9c	748.2±111.9c	242.0±40.5ab	30.2±3.6bc	2410.3±29.9b
	RM	1631.5±33.6b	887.0±25.0a	242.0±7.6ab	33.1±2.7 a	2793.6±59.3 a
	DM	1653.6±75.9b	835.6±82.5b	230.5±28.3b	29.0±6.4 c	2748.6±42.8a
	RSM	1841.8±22.2a	858.9±43.5ab	134.9±3.5c	26.7±2.2b	2862.2±24.0a
	DSM	1800.1±44.7a	894.5±53.9a	139.5±105c	30.5±0.5d	2864.7±35.9a
15–30	RS	1284.6±40.2c	474.0±14.9c	178.1±10.7c	20.8±1.7a	1957.6±46.1d
	DS	1175.0±52.4d	473.8±26.5c	342.8±14.6a	20.0±2.8ab	2011.6±66.2cd
	RM	1402.4±85.2b	469.7±23.1c	203.9±10.6b	20.7±1.1a	2096.6±94.4bc
	DM	1304.4±50.0c	500.7±24.6b	339.3±7.9a	19.1±1.3b	2163.3±45.4b
	RSM	1384.7±28.1b	521.9±20.6b	200.9±16.8b	20.4±0.9a	2127.9±51.6b
	DSM	1510.2±48.6a	659.3±11.7a	151.6±11.0d	17.3±1.6c	2338.4±47.3a

RS, RM, and RSM represent rotary tillage with straw, manure, and straw plus manure application, respectively. DS, DM, and DSM represent deep tillage with straw, manure, and straw plus manure application, respectively. Error bars represent standard deviation of the mean (n = 3). Different lowercase letters indicate significant differences between the treatments at p< 0.05 by the ANOVA, LSD.

In the 15–30 cm soil layer, deep tillage with single organic amendments reduced SOC and TN stocks in >2 mm aggregates by 6.12–7.87% and 6.99–8.53%, respectively, and in 0.25–2 mm aggregates by 5.35–5.77%. By contrast, deep tillage enhanced SOC and TN stocks in 0.053–0.25 mm aggregates by 80.01–89.43% and 66.43–92.45%, respectively.

When straw plus manure was applied, deep tillage exerted a contrasting effect: SOC and TN stocks in >2 mm aggregates increased by 10.49% and 9.07%, and in 0.25–2 mm aggregates by 26.89% and 26.32%, respectively, compared with rotary tillage. Meanwhile, deep tillage reduced SOC stocks in 0.053–0.25 mm aggregates and<0.053 mm clay fractions by 22.16% and 24.54%, respectively. A similar decline was observed in TN stocks of these fractions, with reductions of 8.00% and 15.28%, respectively.

### 3.4 Relationship between wheat yield and soil aggregate-associated carbon and nitrogen stocks

Based on the six-year yield data, the combined application of straw and manure increased wheat yield compared to the application of single organic amendments (**Table 5**). In the first and second years of the experiment, no significant differences in wheat yield were observed among the treatments. In the third and fourth years, the DSM treatment resulted in the highest wheat yield, while no significant differences were detected among the other treatments. In the fifth and sixth years, under single organic amendment application, deep tillage reduced yield by 5.38–7.53% compared with rotary tillage. In contrast, under straw plus manure application, deep tillage increased yield by 8.75–9.58% relative to rotary tillage.

**Table 5:** Effects of different treatments on wheat yield (kg ha^−1^).

Treatment	2017	2018	2019	2020	2021	2022
RS	3512±114a	3556±184a	3799±84b	3552±97c	3885±69c	4233±85b
DS	3569±132a	3616±129a	3913±70b	3647±57c	3613±52d	3959±46c
RM	3642±208a	3700±98a	3949±10b	3628±48c	3958±103bc	4304±94b
DM	3673±162a	3755±184a	4269±22a	3804±69bc	3756±39d	4212±95b
RSM	3657±112a	3712±121a	4120±97ab	3986±87b	4128±124b	4269±51b
DSM	3684±98a	3760±89a	4275±110a	4235±56a	4489±95a	4678±75a

**Figure 6:**
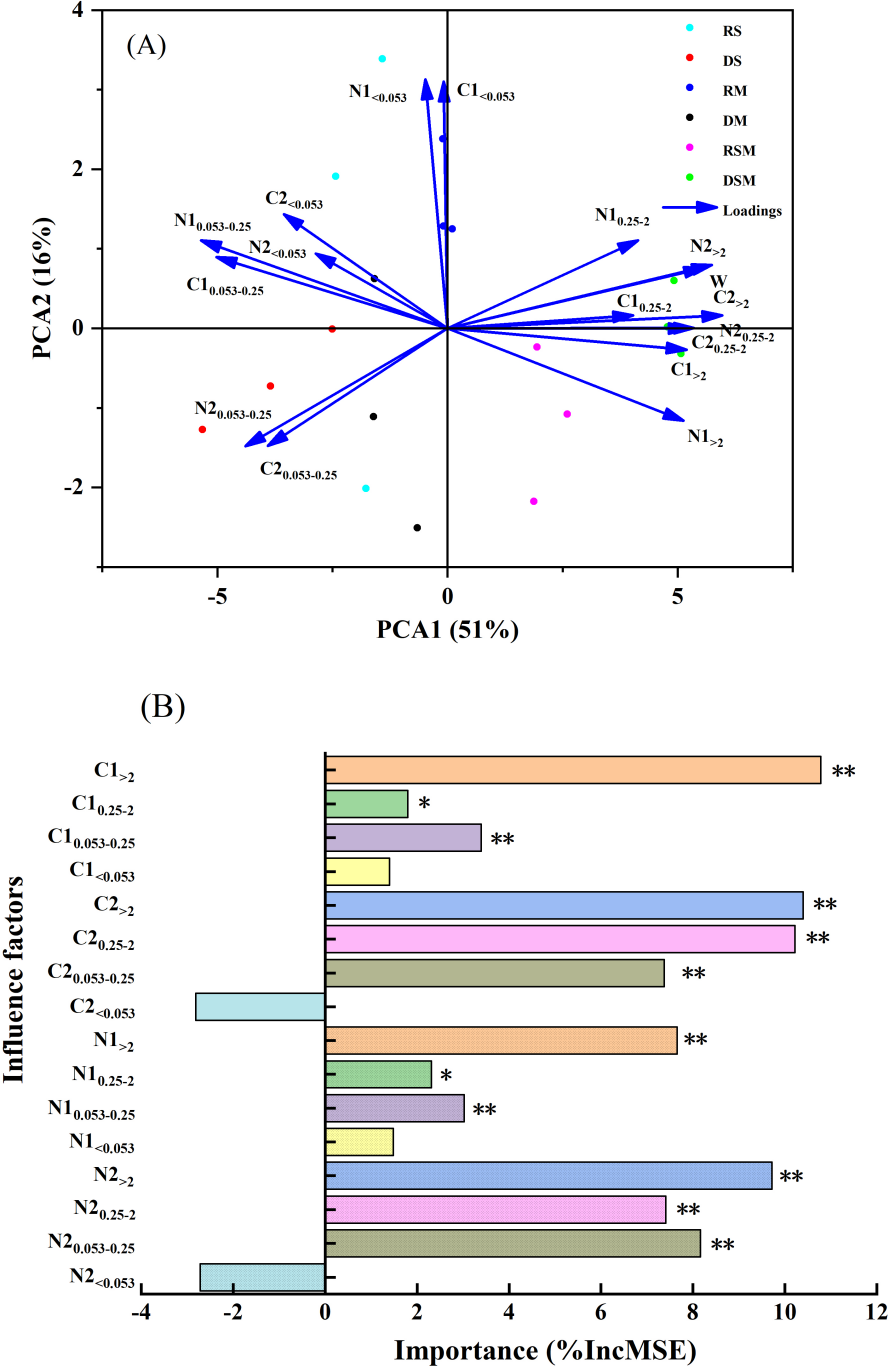
Factors influencing wheat yield. **(A)** The influence of soil organic carbon and total nitrogen stocks in soil aggregate fractions based on principal component analysis (PCA). **(B)** The importance of aggregate-associated organic carbon and total nitrogen stocks based on random forest model. RS, Rotary tillage with straw retention. RS, RM, and RSM represent rotary tillage with straw retention, manure addition, and straw plus manure application, respectively. DS, DM, and DSM represent deep tillage with straw retention, manure addition, and straw plus manure application, respectively. C1 and N1 represent aggregate-associated organic carbon and total nitrogen stocks in the 0–15 cm soil layer, respectively. C2 and N2 represent aggregate-associated organic carbon and total nitrogen stocks in the 15–30 cm soil layer, respectively. The subscripts >2, 0.25-2, 0.053-0.25, and<0.053 represent soil aggregate sizes. W represents wheat yield.

**Figure 7:**
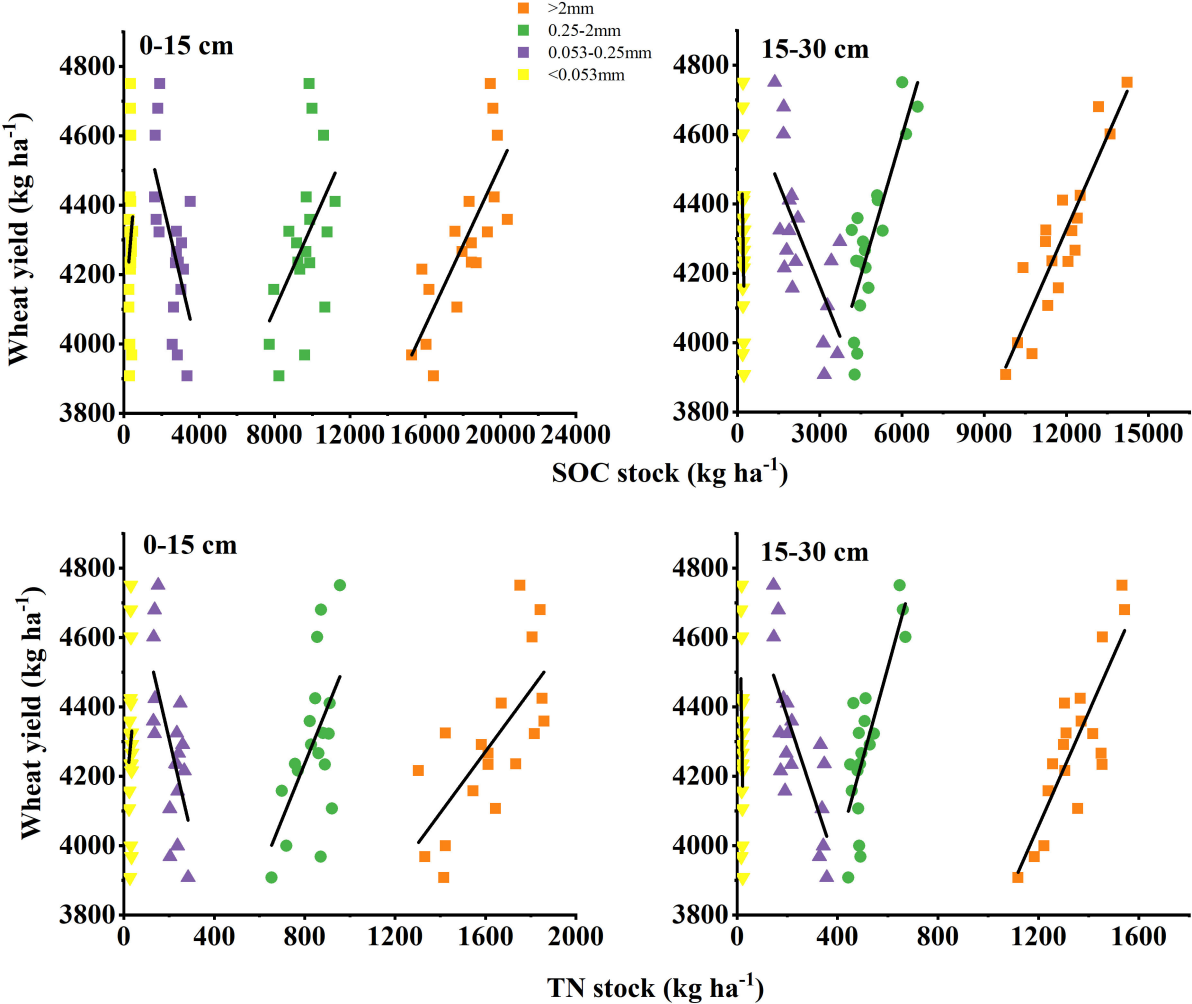
Relationship between wheat yield and soil aggregate-associated organic carbon (SOC) and total nitrogen stocks (TN).

Principal component analysis (PCA) revealed that the first two components (PCA1 and PCA2) explained 51% and 16% of the total variance in wheat yield, respectively (**Figure 6A**). SOC and TN stocks associated with >2 mm and 0.25–2 mm aggregates in both soil layers were the primary contributors to the observed yield variability. Complementary random forest analysis identified SOC and TN stocks in >2 mm aggregates of the 0–15 cm soil layer, as well as SOC and TN stocks in >2 mm and 0.25–2 mm aggregates of the 15–30 cm soil layer, as the most influential predictors of wheat yield (**Figure 6B**). Further validation using linear regression and correlation analysis confirmed that wheat yield was significantly and positively correlated with SOC and TN stocks in >2 mm aggregates (p< 0.01) and 0.25–2 mm aggregates (p< 0.05) of the 0–15 cm soil layer (**Figure 7**; **Table 6**). Similarly, strong positive correlations (p< 0.01) were observed between wheat yield and SOC and TN stocks in >2 mm and 0.25–2 mm aggregates of the 15–30 cm soil layer. In contrast, wheat yield was significantly negatively correlated with SOC and TN stocks in 0.053–0.25 mm aggregates across soil layers (p< 0.01), while no significant relationship was found with SOC and TN stocks in the<0.053 mm clay fraction.

**Table 6:** Regression parameters for wheat yield (*y*) and soil aggregate-associated organic carbon (SOC) and total nitrogen (TN) stocks (*x*).

Soil layer (cm)	Aggregate size (mm)	SOC stock			TN stock		
		Linear equation	*R^2^*	*P*	Linear equation	*R^2^*	*P*
0–15	>2	*y* = 0.1164*x*+2190.23	0.6153	1.16E-04	*y* = 0.8850*x*+2855.59	0.5024	9.90E-04
	0.25–2	*y* = 0.1223*x*+3121.61	0.2065	0.0304	y =1.6103x+2948.54	0.3393	0.0112
	0.053–0.25	*y* = -0.2291*x*+4878.60	0.3835	0.0061	y = -2.7906x+4866.92	0.4240	0.0034
	<0.053	*y* = 0.6468*x*+4065.01	0.0222	0.5548	*y* = 6.6960*x*+4090.85	0.0142	0.6376
15–30	>2	*y* = 0.1796*x*+2171.08	0.8222	2.13E-07	*y* = 1.6442*x*+2083.26	0.7033	1.38E-05
	0.25–2	*y* = 0.2691*x*+2981.96	0.6997	1.52E-05	*y* = 2.6373*x*+2930.08	0.6559	4.64E-05
	0.053–0.25	*y* = -0.1968*x*+4753.73	0.4713	0.0017	*y* = -2.1942*x*+4810.38	0.5696	2.95E-04
	<0.053	*y* = -4.7607*x*+5266.32	0.1348	0.1340	*y* = -45.6703*x*+5192.05	0.1417	0.1236

## 4 Discussion

### 4.1 Soil aggregate size distribution as affected by tillage and organic amendments

Soil aggregates, the fundamental units of soil structure, are highly responsive to agricultural management practices (Dai et al., 2024; Mikha et al., 2015; Xiao et al., 2019). Both tillage operations and organic inputs alter aggregate size distribution and stability by modifying soil physicochemical conditions and microbial activity (Erktan et al., 2020; Mikha et al., 2015). Aggregate stability is commonly assessed by the mean weight diameter (MWD) and geometric mean diameter (GMD), with higher values indicating more stable soil structure (Hu et al., 2021; Yan et al., 2019). In this study, six years of straw plus manure application significantly increased the proportion of >2 mm aggregates and improved GMD values in the 0–15 cm soil layer, compared with straw retention or manure addition alone. This demonstrates the synergistic role of combined organic amendments in promoting macro-aggregate formation and stabilizing topsoil structure in rice–wheat rotations. The macro-aggregates significantly enhance soil structural stability, and it vital for maintaining the sequestration of organic carbon and nitrogen (Guo et al., 2010; Kan et al., 2020; Li et al., 2021).

Deep tillage under single organic inputs reduced the proportions of >2 mm and 0.25–2 mm aggregates in the 15–30 cm soil layer, accompanied by decreases in MWD and GMD. This confirms that deep tillage, despite increasing the plow layer thickness, causes more extensive disruption of subsoil structure than rotary tillage and impairs macro-aggregate formation (Dai et al., 2024). However, when straw and manure were combined, deep tillage enhanced the proportion of 0.25–2 mm aggregates without reducing the abundance of >2 mm aggregates or aggregate stability indices. The organic substrates provided by straw and manure likely stimulated microbial activity, particularly fungal hyphae, which release extracellular polysaccharides and other binding agents that promote soil particle aggregation (Agnihotri et al., 2022; Bu et al., 2020; Lauducina et al., 2015; Shu et al., 2016; Zhou et al., 2021). These findings suggest that the dispersive effects of deep tillage on subsoil aggregates cannot be mitigated by single organic inputs, but a combined input of straw and manure supplies sufficient binding agents to support macro-aggregate formation even under intensive disturbance.

### 4.2 Soil aggregate-associated carbon and nitrogen contents influenced by tillage and organic inputs

Long-term application of straw and organic manure contributed to increased soil carbon and nitrogen nutrient contents. Our findings indicated that (**Table 7**) in the 0–15 cm soil layer, the combined application of straw and manure significantly enhanced soil organic carbon (SOC) and total nitrogen (TN) contents compared with single organic amendments. In the 15–30 cm soil layer, deep tillage increased SOC and TN contents relative to rotary tillage, with the DSM treatment showing the most pronounced improvements in both SOC and TN contents.

**Table 7:** Soil fertility indices under different treatments (g kg^-1^).

Soil layer (cm)	Treatment	SOC content			TN content		
		2018	2020	2022	2018	2020	2022
0—15	RS	14.53c	15.75c	16.58b	1.51b	1.59b	1.61b
	DS	14.80bc	16.02c	15.74c	1.49b	1.56b	1.51c
	RM	15.36a	16.51bc	16.83b	1.55ab	1.61ab	1.68b
	DM	15.37a	16.94ab	16.06bc	1.52b	1.63ab	1.55c
	RSM	15.88a	17.76a	17.98a	1.55ab	1.69a	1.76a
	DSM	16.01a	17.13ab	17.90a	1.59a	1.64ab	1.72a
15—30	RS	8.22b	8.34c	8.47d	0.86c	0.82d	0.97d
	DS	8.54b	8.80c	8.95c	0.97b	0.90c	1.10c
	RM	8.43b	8.72c	8.66cd	0.95b	1.10b	1.14c
	DM	8.67b	9.52b	9.52b	0.98b	1.19a	1.21b
	RSM	8.61b	9.36b	9.81b	1.01b	1.15b	1.16c
	DSM	9.29a	10.19a	10.97a	1.14a	1.21a	1.27a

The formation and stabilization of aggregates are closely linked to SOC and TN dynamics, as aggregates provide physical protection for organic matter, while SOC and TN availability in turn influence aggregate development (Curtin et al., 2014; Fraser et al., 2013; Wang et al., 2017). Organic amendments such as straw and manure are rich sources of carbon and nitrogen, and their long-term incorporation enhances SOC and TN contents across aggregate fractions (Hansen et al., 2016; Li et al., 2018; Tian et al., 2022). Our findings showed that combined straw and manure inputs significantly increased SOC and TN contents in macro-aggregates (>2 mm and 0.25–2 mm) compared to single organic inputs. Deep tillage further increased SOC and TN contents in macro-aggregates within the 15–30 cm soil layer, likely due to the thorough incorporation of organic materials into deeper horizons. While rotary tillage tends to confine organic matter to the surface, deep tillage facilitates vertical mixing, accelerating decomposition and nutrient incorporation in subsoil aggregates (Barreto et al., 2009; Dikgwatlhe et al., 2014; Guan et al., 2015). These results highlight the importance of both tillage depth and the type of organic input in regulating aggregate-associated nutrient pools, particularly in subsoil layers.

### 4.3 Soil aggregate-associated carbon and nitrogen stocks as influenced by tillage and organic amendments

Macro-aggregates are recognized as major sinks of soil carbon and nitrogen, contributing to long-term stabilization of organic matter (Lehmann and Kleber, 2015). In the present study, straw plus manure application consistently enhanced SOC and TN stocks in macro-aggregates of the 0–15 cm layer compared to single organic inputs. The likely explanation is that the combined input of straw and manure supplies a greater diversity and quantity of organic substrates, increasing the availability of both energy sources and binding agents for aggregate formation.

In the subsoil (15–30 cm), deep tillage reduced SOC and TN stocks in macro-aggregates under single organic amendments, despite higher SOC and TN contents. This indicates that aggregate disruption outweighed the nutrient input effect, leading to a net decline in macro-aggregate-associated stocks (Ashagrie et al., 2007; Li et al., 2024). By contrast, under straw plus manure application, deep tillage significantly increased SOC and TN stocks in macro-aggregates. The combined amendments not only elevated C and N availability but also supplied sufficient cementing agents to support macro-aggregate formation, thereby offsetting the structural disruption caused by deep tillage (Alcantara et al., 2016; Hao et al., 2019; Hansen et al., 2016; Lauducina et al., 2015). These findings emphasize that the interaction between tillage depth and organic inputs governs the balance between aggregate destruction and re-formation, ultimately determining SOC and TN sequestration in different soil layers. Future research will systematically collect time-series data on SOC and TN at the aggregate scale. By monitoring the dynamics of aggregate turnover and the speciation of SOC and TN, we aim to further elucidate the underlying mechanisms driving soil carbon and nitrogen sequestration. This work will deepen our understanding of how tillage depth and organic material inputs influence the processes of SOC and TN stabilization in soils.

### 4.4 Linking soil aggregate-associated carbon and nitrogen stocks to wheat yield

Sustainable crop production relies on favorable soil structure and sufficient SOC and TN stocks, particularly within macro-aggregates that regulate nutrient supply and soil fertility (Bu et al., 2023; Ma et al., 2023). In this study, DSM treatment (deep tillage with straw plus manure) produced the highest wheat yield, reflecting improvements in aggregate stability and increases in macro-aggregate-associated SOC and TN stocks. Correlation analysis confirmed that wheat yield was strongly and positively associated with SOC and TN stocks in macro-aggregates, especially in the 15–30 cm layer.

This finding highlights the pivotal role of subsoil macro-aggregates in supporting crop productivity. Enhanced nutrient storage and release from these aggregates likely improved the soil’s capacity to sustain wheat growth and yield (Liu et al., 2025; Tian et al., 2022; Wang et al., 2023; Kan et al., 2025). Thus, integrating deep tillage with combined organic amendments provides a strategy for enhancing soil structural quality and nutrient pools in both surface and subsoil layers, thereby improving yield potential in rice–wheat rotation systems.

Although strong correlations between soil macro-aggregate C/N stocks and wheat yield were observed in this study, future research employing techniques such as isotopic tracing or path analysis is still required to definitively establish direct causal links.

## 5 Conclusions

**Figure 8:**
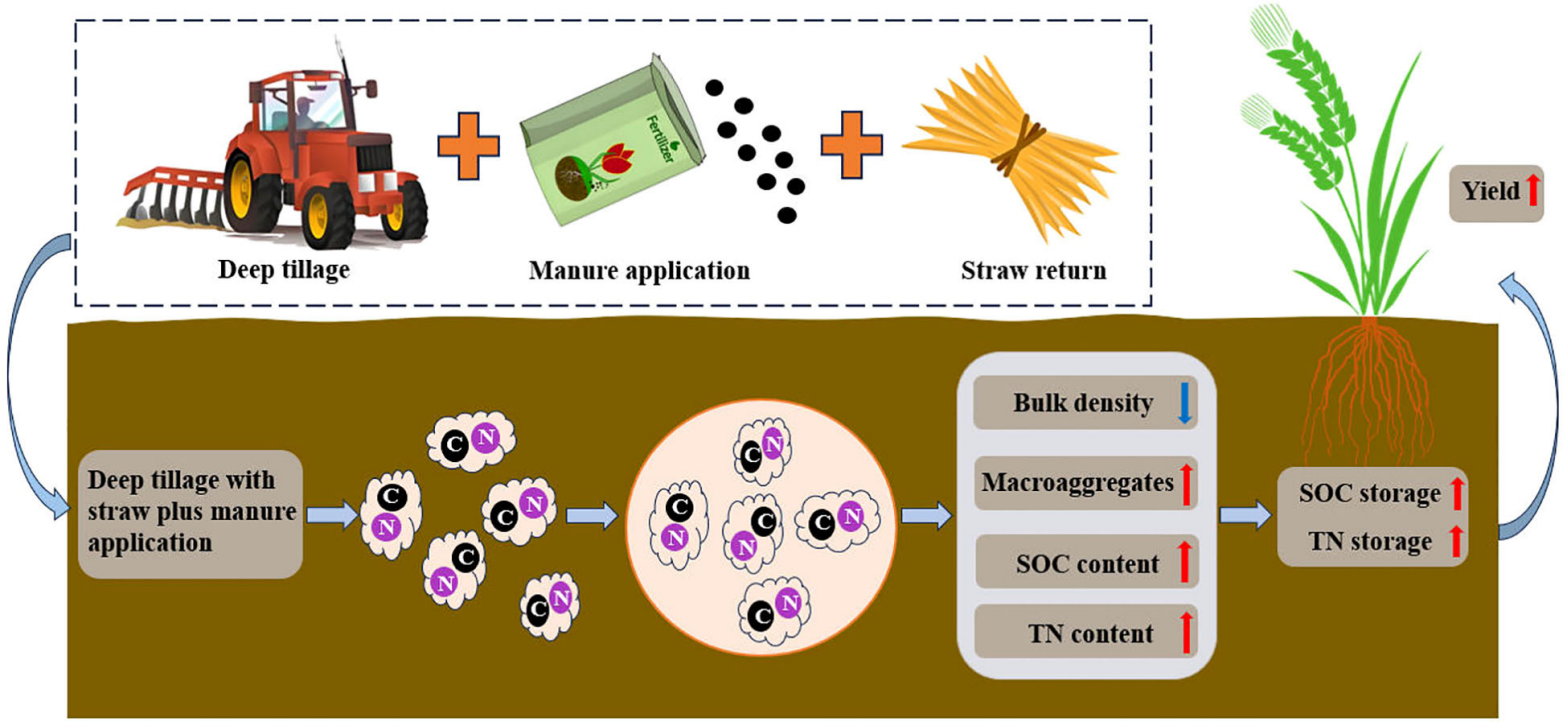
Conceptual diagram of the effects of deep tillage combined with organic amendments on carbon and nitrogen storage within aggregates and wheat yield.

This study demonstrated that tillage depth and the type of organic amendment interactively regulate soil aggregate structure, carbon and nitrogen sequestration, and wheat yield in a rice–wheat rotation system (**Figure 8**). Deep tillage increased SOC and TN contents in macro-aggregates of the subsoil but simultaneously reduced their proportions under single organic inputs, resulting in lower macro-aggregate-associated SOC and TN stocks. In contrast, the combined application of straw and manure offset the disruptive effect of deep tillage on aggregate stability, enhanced SOC and TN contents in macro-aggregates, and thereby improved aggregate-associated nutrient stocks. Enhanced macro-aggregate-associated SOC and TN stocks were strongly and positively correlated with wheat yield, particularly in the 15–30 cm soil layer, underscoring the critical role of subsoil nutrient storage in sustaining crop productivity. These results highlight deep tillage with straw plus manure as an effective management practice to enhance soil structural stability, increase subsoil C and N stocks, and improve wheat yield in rice–wheat cropping systems. Future research will systematically integrate the characteristics of soil carbon/nitrogen stocks and yield under rice stubble conditions, conducting in-depth exploration of the long-term ecological effects of tillage and fertilization practices from an annual cycle perspective. This approach holds significant importance for unveiling the complete picture of carbon and nitrogen cycling in rice-wheat rotation systems.

## Data Availability

The data analyzed in this study is subject to the following licenses/restrictions: The data are available on request from the authors. The data are not publicly available due to confidentiality agreements. Requests to access these datasets should be directed to Wenlong Cheng, wlchengche@163.com.
